# Assessment of the American College of Surgeons Surgical Risk Calculator (ACS-SRC) for Prediction of Early Postoperative Complications in Patients Undergoing Cytoreductive Surgery for Ovarian Peritoneal Carcinomatosis

**DOI:** 10.3390/curroncol31120579

**Published:** 2024-12-07

**Authors:** Cedric Kabeya, Charif Khaled, Laura Polastro, Michel Moreau, Dario Bucella, Maxime Fastrez, Gabriel Liberale

**Affiliations:** 1 Department of Digestive Surgery and Digestive Surgical Oncology, Jules Bordet Institute, The Brussels University Hospital (H.U.B), Université Libre de Bruxelles (ULB), Meylemeersch Street 90, 1070 Brussels, Belgium; cedrickabeya@outlook.be (C.K.); charif.khaled@hubruxelles.be (C.K.); 2 Department of Medical Oncology, Jules Bordet Institute, The Brussels University Hospital (H.U.B), Université Libre de Bruxelles (ULB), Meylemeersch Street 90, 1070 Brussels, Belgium; laura.polastro@hubruxelles.be; 3 Department of Statistics, Jules Bordet Institute, The Brussels University Hospital (H.U.B), Université Libre de Bruxelles (ULB), Meylemeersch Street 90, 1070 Brussels, Belgium; michel.moreau@hubruxelles.be; 4 Department of Gynecology, CHU Saint-Pierre, Université Libre de Bruxelles (ULB), Rue aux Laines 105, 1000 Brussels, Belgium; dario.bucella@stpierre-bru.be; 5 Department of Gynecology, Jules Bordet Institute, The Brussels University Hospital (H.U.B), Université Libre de Bruxelles (ULB), Meylemeersch Street 90, 1070 Brussels, Belgium; maxime.fastrez@hubruxelles.be

**Keywords:** ovarian cancer, peritoneal metastasis, cytoreductive surgery, ACS-SRC, postoperative complications, predictive calculator

## Abstract

Ovarian cancer (OC) is diagnosed at a locally advanced stage in two-thirds of cases. The first line of treatment consists of cytoreductive surgery (CRS) combined with neoadjuvant and/or adjuvant chemotherapy. However, CRS can be associated with high rates of postoperative complications (POCs), and detection of fragile patients at high risk of POCs is important. The American College of Surgeons Surgical Risk Calculator (ACS-SRC) provides a predictive model for early POCs (30 days) for any given surgical procedure. This study aimed to evaluate the performance of the ACS-SRC in predicting the occurrence of early POCs for patients undergoing CRS for OC. This was a retrospective study that included patients undergoing CRS for advanced OC between January 2010 and December 2022. Early POCs were reviewed, and the rate of POCs was compared with those predicted by the ACS-SRC to evaluate its accuracy (i.e., discrimination and calibration). A total of 218 patients were included, 112 of whom underwent extensive surgery/resection. A total of 94 complications were recorded. This cohort demonstrated correct calibration of the ACS-SRC for the prediction of surgical site infection, readmission, and the need for nursing care post-discharge (NCPD; transfer to revalidation center or need for nursing care at home). Using both the discrimination and calibration methods, the score only predicted NCPD. In this study, the ACS-SRC was shown to be of little value for patients undergoing cytoreductive surgery for ovarian peritoneal carcinomatosis, as it only accurately predicted NCPD.

## 1. Introduction

Ovarian cancer (OC) is the second most common pelvic gynecological cancer [1] and accounts for around 30% of gynecologic malignancy [2]. It represents 1% of all new cancer cases but accounts for 2% of all cancer deaths, with an estimated survival at 5 years of 50% [3]. This is because 75% of cases are diagnosed at an advanced stage [4]. The clinical presentation of OC is often nonspecific, consisting of abdominal discomfort, painful bloating, bowel movement disorders, and distension due to the presence of ascites [1]. Epithelia OC (EOC) is the most common histologic type, accounting for more than 90% of the cases. It takes origin from the epithelial cells and can be either high-grade serous, low-grade serous, endometrioid, clear cell, or mucinous carcinoma [2,5]. While BRCA 1 and 2 mutations are the most common predisposing factors for EOC, mutations are only found in 15–20% of patients [6].

In locally advanced OC, cytoreductive surgery (CRS) is performed via a xypho-pubic median laparotomy and involves resection of all macroscopically visible lesions within the peritoneal cavity (completeness of cytoreduction 0: CC-0) [7]. CRS can either be upfront, followed by six courses of carboplatin-paclitaxel chemotherapy [8,9], or at an interval stage, following neoadjuvant chemotherapy (NAC). This second option is proposed in high-burden peritoneal disease reflected by a high Peritoneal Cancer Index (PCI) score [1,7] and/or a Fagotti score > 2 at first exploration [10]. According to the literature, reported CRS postoperative complication (POC) rates vary from 15% to 25%, and mortality rates are around 3% [11,12,13,14]. Therefore, fragile patients at a high risk of severe POCs need to be properly identified to ensure a favorable postoperative outcome and maximize the benefit of surgery in terms of survival outcomes.

The American College of Surgeons-Surgical Risk Calculator (ACS-SRC) estimates the risk of early POCs (within 30 days) based on 21 patient characteristics [15]. The ACS-SRC was constructed using a database of more than five million procedures, reported as “Current Procedural Terminology (CPT)” codes, among 874 participating hospitals between 2016 and 2020 [15]. Of these procedures, 7000 cases involved surgeries for OC [16]. The ACS-SRC was shown to be an adequate predictor for severe POCs after curative surgery for abdominal tumors [17]. However, to our knowledge, only one study has evaluated the performance of the ACS calculator in the prediction of POCs for OC patients [18]. That study only included patients undergoing interval CRS and reported that the ACS calculator was unable to accurately detect which patients were at risk for POCs and longer hospital stays, with very limited preoperative use for CRS for OC.

The aim of this study was to further evaluate the performance of the ACS-SRC in predicting early POCs by including patients undergoing upfront or interval CRS for advanced OC.

## 2. Materials and Methods

### 2.1. Study Design and Patients

This was a retrospective study at the Jules Bordet Institute and Erasme Hospital, both part of the Brussels University Hospital (H.U.B), Université Libre de Bruxelles (ULB). It included patients who underwent open CRS for advanced OC for primary or recurrent disease from 1 January 2010 to 31 December 2022. Ethical committee approval was obtained prior to the beginning of the study.

Patient inclusion criteria were age > 18 years of age; tubo-ovarian or peritoneal epithelial carcinoma; and CRS for locally advanced primary or recurrent cancer (stage III or IV according to the classification of the International Federation of Gynecology and Obstetrics (FIGO)).

Patients with incomplete medical records were excluded.

### 2.2. Cytoreductive Surgery (CRS)

CRS typically consisted of total radical hysterectomy with bilateral salpingo-oophorectomy, complete omentectomy (infra- and supra-colonic), and resection or electrofulguration of any suspected peritoneal nodules/scars/fibrosis visible during exploration. Pelvic and lumbo-aortic lymphadenectomy were frequently performed in patients before 2019, regardless of preoperative lymph node status; however, in recent years, this has become dependent on clinical FIGO staging and preoperative imaging [19]. In patients who underwent multiple surgeries, the procedure chosen for analysis was the first one performed at our hospital (whether for primary or recurrent disease). We used the CC score to refer to the completeness of cytoreduction, with CC-0 meaning no macroscopic residual disease.

### 2.3. Data and ACS-SRC

The following data were collected from multidisciplinary oncology committee reports, surgical consultations, anesthesia consultations, and medical imaging protocols:

- Demographic and clinical characteristics: age, gender, body mass index (BMI), type of procedure (CPT code), functional status of the patient (daily third-party dependency);
- General preoperative status and medical conditions: American Society of Anesthesiology (ASA) score, chronic corticosteroid therapy, diabetes, hypertension (HTN), heart failure within 30 days prior to surgery, dyspnea, smoking within one year prior to surgery, chronic obstructive pulmonary disease (COPD), acute and chronic renal failure (RF), dialysis, presence of ascites during the 30 days prior to surgery, sepsis during the 48 h prior to surgery, oxygen dependency prior to surgery;
- FIGO stage and other oncologic data (e.g., chemotherapy, date of diagnosis, date of surgery);
- POCs were determined based on laboratory analyses, imaging, intensive care unit and surgical ward follow-up notes, and consultation notes in the 30-day postoperative period and classified according to the Clavien-Dindo (CD) classification.

### 2.4. ACS-SRC: Calculating the Risk of POCs

The ACS-SRC was used for each patient included in the study; the above data were manually entered into the ACS-SRC online website (https://riskcalculator.facs.org, accessed between July 2023 and December 2023) and the estimated risk percentage of each complication predicted by the calculator was collected. The list of POCs predicted by the ACS-SRC included pneumonia, cardiac complications (e.g., cardiac arrest or myocardial infarction), surgical site infection (SSI), urinary tract infection (UTI), venous thromboembolism (VTE), RF, readmission, re-intervention, death, and prolonged hospital stay.

The ACS-SRC includes a section for encoding one CPT procedure. For each patient, the most appropriate CPT code was used. For simple debulking with no resection and no lymphadenectomy, CPT 58956 or CPT 58953 was used. For simple debulking associated with lymphadenectomy, CPT 58951 or CPT 58954 was used. In cases of recurrent disease, CPT 58957 or CPT 58958 was used. In cases with digestive resections, CPT 38102 or CPT 44120 or CPT 44145 or CPT 47120 was used.

CRS frequently involves several surgical procedures during the same operation. In the ACS-SRC, it is impossible to simultaneously encode several procedures. Therefore, to evaluate the best manner for predicting POCs, three calculation methods for each patient were performed as follows:

- First, we exclusively encoded CPT for ovarian debulking (5895X), distinguishing between standard or radical surgery, primary or secondary surgery (for recurrence), and surgery with or without lymph node dissection.
- Second, we integrated the above-mentioned CPT as well as CPT for digestive surgery [20]. In the case of digestive resection, the calculator was run twice, and the highest score per complication was used.
- Third, we used the adjustment score proposed by the site on the scores obtained after the introduction of ovarian debulking CPT (risk 1 to 3). Procedures involving only the usual resections (i.e., hysterectomy with salpingo-oophorectomy, omentectomy, and resection of peritoneal carcinomatosis nodules) were classified as risk 1. Procedures involving a resection considered by the surgical team to be of moderate complexity (i.e., splenectomy, enlarged peritonectomy of the Douglas and/or diaphragm, small bowel resection) were classified as risk 2. Those with a resection of complexity considered important and at high risk of complications by the surgical team (i.e., colectomy, rectal resection) were classified as risk 3 [13,14].

### 2.5. Statistical Analysis

Statistical analyses were performed using SAS 9.4 by the institution’s biostatistician. Analysis included a descriptive analysis of the study population and their characteristics expressed as mean (±standard deviation) and proportion (in %), depending on whether the variables were continuous or discrete. Discrete variables were evaluated using the chi-squared test, and continuous variables were evaluated using Student’s *t*-test.

The predictive performance of the ACS-SRC was analyzed by assessing discrimination and calibration. Discrimination measures the ability of a regression model to differentiate a patient at high risk for a given event (e.g., cardiac complication) from others, using a set of predictor variables for the given event (e.g., BMI, age, co-morbidities). This makes it possible to characterize the risk of one patient compared to another, without considering risks in absolute values. Calibration measures the ability of a model to assign an absolute value to an estimated risk, making it possible to analyze whether the absolute value of observed events is statistically comparable to the absolute value of events estimated by the model [21].

In this study, discrimination was analyzed by calculating the area under the curve (AUC) of a Receiver Operating Characteristic (ROC) graph. This statistical model quantifies the association between the estimated risk and the observed POC in the patient. The AUC values are in the range of 0 to 1. For example, a model with a 40% chance of a right prediction has an AUC of 0.4. Discrimination is considered poor for an AUC between 0.6 and 0.69, acceptable if between 0.7 and 0.79, powerful if between 0.8 and 0.89, and excellent if between 0.9 and 1.0.

The Brier score simultaneously evaluates discrimination and calibration [12,20,22], a criterion that provides combined information on the accuracy of the prediction. It describes the mean square difference between a predicted risk and the actual outcomes, making it an effective metric for evaluating the accuracy of a prediction model: If the probability of a complication occurring is 100% and the patient has this complication, the score is 0, and the predictive model is considered perfect. If the probability of a complication is 100% but the patient does not have this complication, the score is 1, and the model is considered unreliable. If the probability of a complication is 50%, the score is 0.25. An estimated risk with a Brier score greater than or equal to 0.25 is considered non-informative [23,24]. In this cohort, the calculator was considered reliable if the Brier score < 0.01, indicating a high level of predictive accuracy. Statistical analyses are considered significant if the *p*-value < 0.05.

## 3. Results

**Figure 1:**
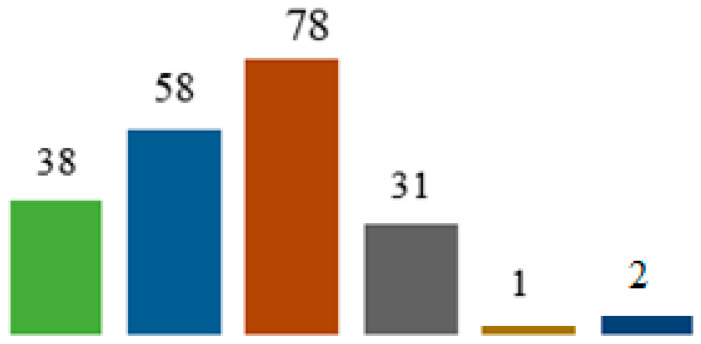
Patient distribution by Current Procedural Terminology (CPT) code. 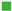 Debulking + lymphadenectomy, 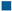 debulking + resection, 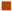 debulking + resection + lymphadenectomy, 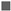 debulking, 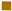 debulking for recurrent disease, and 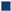 debulking for recurrent disease + lymphadenectomy.

A total of 267 OC patients underwent surgery at the included institutions between 2010 and 2022. Of these, 21 patients were excluded for FIGO I–II, 23 patients were excluded for incomplete surgery, and 5 patients were excluded for missing data. Finally, 218 patients were included in the study, 77% of whom received NAC. Patient characteristics are reported in Table 1. Figure 1 shows the distribution of patients according to CPT code. The median age was 63 years, with a mean BMI of 25 kg/m^2^. Hypertension was present in 38% of cases, smoking in 10%, and diabetes in 9%. One hundred fifty-six patients (71.6%) were ASA II. In 112 patients (51%), surgery was considered extensive (i.e., including digestive/splenic resections and/or extended peritonectomies). Of these 112 patients, 97 had digestive resections + anastomosis. The median postoperative stay was 12 days. Concerning CRS, 89% of resections were CC-0, 3% CC-1, and 8% CC-2.

### 3.1. Complications

Ninety-four patients (43%) developed one or more early POCs (totaling 142 complications). Of those, 31 patients (14%) had major complications (Clavien-Dindo (CD) grade III–IV). The mortality rate was 0.5% (one patient who had sepsis and developed pulmonary effusion and respiratory arrest followed by cardiac arrest on postoperative day 12). In order of frequency, UTI (n = 45), intra-abdominal abscess (n = 12), and bacteremia (n = 12) accounted for 48%, 13%, and 13% of complications, respectively.

By convention, in the univariate analysis, the predetermined minimum number of events required for interpretation is ten. Among the outcomes evaluated by the ACS-SRC, the incidences of cardiac complications (n = 6), VTE (n = 3), RF (n = 6), and death (n = 1) did not reach this minimum threshold and, therefore, are considered as uninterpretable.

According to the three calculations performed for each patient, the prediction of POCs proposed by the calculator underestimated the incidence of all complications observed in our cohort with the exception of readmission rate.

#### 3.1.1. Discrimination

In terms of discrimination, with method n°1, the calculator showed significant results for NCPD (c-statistic = 0.70) and death* (Brier score = 0.004). With method n°2, the calculator only showed that death* was well discriminated (Brier score = 0.004). Finally, with method n°3, the calculator showed significant results for NCPD (c-statistic = 0.71) and death* (Brier score = 0.005). Note that *death was mentioned for information purposes only (given the insufficient number of events).

#### 3.1.2. Calibration

In terms of calibration, with method n°1 (CPT debulking), SSI (*p* = 0.003) and need of nursing care post-discharge (NCPD; *p* = 0.04) were statistically significant. With method n°2 (double scoring: CPT debulking and resection; highest score), readmission (*p* = 0.009) and NCPD (*p* = 0.02) were statistically significant. Finally, with method n°3 (CPT debulking weighted by risk adjustment), SSI (*p* = 0.005), readmission (*p* = 0.04), and NCPD (*p* = 0.05) were statistically significant. The calculator was not correctly calibrated for the other complications despite the use of three different analysis methods.

## 4. Discussion

This is the first study to evaluate the predictive performance of the ACS-SRC in patients treated with upfront or interval CRS for stage III/IV ovarian cancer. This study suggests that the ACS-SRC underestimates all POCs except NCPD. The calculator, using the main CPT code with or without the adjustment score (risk 1–3), discriminates well and is well calibrated for NCPD. Moreover, the calculator was not able to discriminate patients but was well calibrated to predict SSI (methods 1 and 3) and readmission rates (methods 2 and 3).

The ACS predictive model was developed to help surgeons rationalize surgical indications, taking into account the risk of POCs, particularly in fragile patients. Its validity in gynecological oncology, particularly advanced OC, has not been well evaluated [18,20,23].

The first studies to evaluate the effectiveness of the ACS-SRC calculator on gynecological procedures (benign and malignant combined) were published by Rivard et al. and Szender et al. [20,23]. A more recent study published by Manning et al. in 2021 [18] looked at 261 patients who had exclusively undergone interval ovarian debulking [18]. The results of these three retrospective studies highlight a tendency for the ACS-SRC to grossly underestimate the various complications predicted by the calculator. The overall POC rate in the present study was 43%, with 14% being severe complications (CD III/IV). This is comparable to other studies that have reported CD I/II complications of 20–22% and CD III/IV complications of 11–15% [8,14]. The ACS-SRC failed to predict these rates.

In the present cohort, the calculator demonstrated appropriate discrimination in the prediction of NCPD (c-statistic = 0.70–0.71 depending on the method used). For calibration, it was satisfactory for SSI (*p* = 0.003–0.005), risk of readmission (*p* = 0.04–0.009), and NCPD (*p* = 0.02–0.05). In the aforementioned studies [20,23], the calculator demonstrated satisfactory discrimination with regard to prediction of RF, cardiac complications, pneumonia, VTE, and death, but other predictions of POC were not considered reliable [20,23]. In the present study, the number of events for RF, cardiac complications, VTE, and death was low (<10) and, therefore, those results were not interpreted. In the Manning study, the calculator was not discriminative for any of the proposed outcomes but was appropriately calibrated for hospital length of stay [18].

The ACS calculator has been validated on more than 7000 patients with ovarian cancer [16]. However, this study did not take into consideration the CC and residual disease at the end of the operation. Therefore, it included all types of resections, which would underestimate the results when compared to populations undergoing complete CRS (CC-0/1). Moreover, the population studied by the ACS to elaborate the SRC contained a low proportion of gynecological procedures (1.5%) [12], and the surgeries recorded in the ACS registry are essentially of low complexity [9].

Some of the clinical data proposed by the ACS calculator portal lack precision. For instance, concerning the preoperative presence of ascites, for which the calculator portal does not require volume specification despite the fact that volume has been reported as a predictive factor for postoperative complications [8].

The ACS score also misses other important predictive factors of POCs for CRS. The Peritoneal Cancer Index (PCI) is used to assess the extent of peritoneal carcinomatosis in locally advanced cancer and has been reported to be related to increased POC rate in patients with PCI ≥ 21 [8,13]. In the present cohort, patients who developed pneumonia had significantly higher average PCI values (19 vs. 11, *p* = 0.02), and this was also true for those with VTE (33.5 vs. 12, *p* = 0.004).

Another factor that could potentially influence the risk of POCs after CRS is the degree of radicality with the goal to achieve a CC-0 resection [25]. The extensiveness inherent in the radical nature of this surgery is associated with a higher severe complication rate (OR of 1.96) compared with standard procedures [7,11,16,26].

A more specific predictive model for gynecological oncology, developed in 2019 by Cham et al., that incorporates preoperative albumin and a surgical complexity score, independently validated in 2021, is also not implemented in the calculator [9,16]. Another model was developed by Vizielli et al. [27] based on the most common POC predictors (age, performance status, albuminemia, CA-125, presence of ascites [26]) and tumor load on preoperative laparoscopy. It proved to be somewhat accurate in predicting POC risk [27]. However, their model only included patients undergoing primary CRS.

Finally, this study has several limitations. First, its retrospective design leaves open the potential for missing information concerning POCs. Second, the limited number of patients included in the study impaired the statistical analysis of several complications such as cardiac complications, VTE, RF, and death.

## 5. Conclusions

The use of a calculator to predict POCs in patients with locally advanced OC that need to undergo CRS is important. It helps provide patients with an estimate of the risk and helps surgeons/oncologists to better select candidates for surgery based on the identification of those with a reasonable risk. However, the ACS-SRC calculator does not reliably predict POCs in this setting. The calculator using the main CPT code, with or without the adjustment score, discriminates well and is well calibrated for the prediction of NCPD. Further efforts should be made to refine the calculator for predicting POCs in patients treated for stage III/IV ovarian cancer with complete CRS; this could be achieved by adding certain variables to the score, like volume of ascites, residual disease, and PCI score.

## Figures and Tables

**Table 1:** Population characteristics.

Variable		N (%)
Patients (*n*)		218
Age, years (mean ± SD)		61 ± 12.5
BMI, kg/m^2^ (mean ± SD)		25 ± 5.2
ASA (%)	I	6 (2.8%)
	II	156 (71.6%)
	III	56 (25.7%)
NAC		168 (77%)
Ascites (%)		100 (45.9%)
Diabetes (%)		19 (8.7%)
HTN (%)		83 (38.1%)
Dyspnea (%)		18 (8.3%)
Smokers (%)		22 (10.1%)
Preop albuminemia, g/L (mean ± SD)		41.6 ± 6.13
PCI (mean ± SD)		13.3 ± 10
Hospital stay, days (mean ± SD)		9 ± 7.2
Extensive surgery (%)		112 (51.4%)
Digestive resections		97
Patients with complications (%)		93 (42.7%)
CD I–II		68
CD III–IV		25
Pulmonary (%)		10 (4.6%)
Cardiac		6 (2.8%)
SSI		32 (14.7%)
Anastomotic leaks		4 (1.83%)
UTI		46 (21.1%)
VTE		3 (1.4%)
RF		6 (2.8%)
Sepsis		13 (6%)
Death		1 (0.5%)
Reoperation		18 (8.3%)
Readmission		21 (9.6%)
NCPD		27 (12.4%)

## Data Availability

Research data supporting this publication is available at the editor’s request.
